# Thiol-redox proteins interact with soluble guanylyl cyclase and modulate its activity

**DOI:** 10.1186/2050-6511-14-S1-O21

**Published:** 2013-08-29

**Authors:** Erin J Heckler, Mohit Jain, Pierre-Antoine Crassous, Angelo R Rossi, Hong Li, Annie Beuve

**Affiliations:** 1 Department of Pharmacology and Physiology, New Jersey Medical School, Newark, NJ, USA; 2 Center for Advanced Proteomics Research, New Jersey Medical School Cancer Center, Newark, NJ, USA; 3 High Performance Computing Center, New Jersey Medical School, Newark, NJ, USA

## Background

Nitric oxide (NO) signalling through activation of soluble guanylyl cyclase (sGC) is key for the physiology of the cardiovascular and neuronal systems. Discrepancies in sGC activation and deactivation *in vitro* vs. *in vivo* have led to a search for endogenous factors that regulate sGC or assist in cellular localization. In our previous work, which identified Hsp70 as a modulator of sGC, we determined that protein disulfide isomerase (PDI) bound to an sGC-affinity matrix [1]. As several studies have reported that thiol oxidants and reductants modulate sGC activity, we sought to characterize the potential interaction between sGC and PDI, an enzyme that not only isomerases disulfide bonds but also regulates some proteins activity via interaction with its cysteine (Cys) active sites [2,3].

## Results

Using protein chemistry and purified sGC and PDI, we first showed that reduced sGC was modified by oxidized PDI in non-reducing Western blot and that sGC thiol titer was altered under these conditions. Moreover a PDI-Flag resin retains specifically sGC and this complex was resolved in the presence of a thiol-reductant. These data suggested that the mechanism of interaction between sGC and PDI is by mixed-disulfide exchange. In addition, PDI modulated NO-stimulated sGC activity while a PDI mutated in its two Cys active sites did not. A relatively novel method, *in situ* PLA (proximity ligation assay) established that endogenous PDI and sGC also interact in the CRL 2018 smooth muscle cells suggesting that the interaction between sGC and PDI is biologically relevant. Notably, both α1 and β1 sGC subunits of sGC showed potential co-localization with PDI [4].

## Discussion

These results underlined the significance of redox-thiol modulation of sGC and support past and current research showing that certain sGC Cys are necessary for such functions as enzyme activation, dimerization, and desensitization by S-nitrosation. A novel Mass Spectrometry analysis is currently applied to identify sGC Cys disulfide bonds and combined with mutational analysis and molecular dynamics simulation should unveil their involvement in interaction with PDI and their relevance as a new mechanism of sGC modulation.
